# Complicated Interactions in Home Care: An Ethnographic Study

**DOI:** 10.1177/23333936261426317

**Published:** 2026-02-26

**Authors:** Vilhelmina Th. Einarsdottir, Asta Snorradottir, Jeannette Pols, Holly Symonds-Brown, Kristin Bjornsdottir

**Affiliations:** 1 University of Iceland, Reykjavik, Iceland; 2 University of Amsterdam, The Netherlands; 3 University of Alberta, Edmonton, Canada

**Keywords:** caregivers, caregiving, communication, ethics/moral perspectives, ethnography, interviews, observations, home care nursing, community, relationships, patient-provider, social services, workplace, Iceland

## Abstract

In this qualitative ethnographic study, we aimed at increasing understanding of what makes interactions between staff, users, and relatives in home care services complicated, how these interactions are experienced and how home care practice can be developed to better address these interactions. The study was conducted in Reykjavik, the capital of Iceland, and data were collected from January 2021 to February 2022. In this article we present findings reflecting the experiences of the staff. Home care work involves frequent interactions with service users and their relatives, and the quality of these interactions can influence both care provision and overall well-being. This qualitative ethnographic study aimed to enhance understanding of what makes interactions among staff, service users, and relatives in home care services complex, how these interactions are experienced, and how home care practice might be developed to better address them. The study draws on observations of interactions between staff and service users, as well as semi-structured interviews with service users, relatives, and staff, involving a total of 35 participants. Three themes were developed from the data, informed by the theoretical background (emotional labor and care practices approach). They were: (a) frictions and emotional difficulties, (b) home and workplace, and (c) problems associated with substance use. The findings show that staff need to be aware of how they communicate with users and their relatives and understand the emotional impact of complicated interactions. Managers of Home Care organizations play an important role in supporting and guiding staff concerning complex interactions.

## Introduction

In the coming years, the need for home care services will increase due to population ageing and demographic changes. Consequently, researchers have called for new knowledge and further development of care delivery approaches in home care. Home care services are unique in that they take place in the homes of those who need care. Compared to institutional health care settings, the home is a private territory that staff enter with the aim of providing care. In this way the home may become a semi-public space, although the owner remains in charge. Staff experience themselves as both guests and professionals (Fjørtoft et al., 2021; Öresland et al., 2008). In a scoping review, Dostálová et al. (2021) report evidence that some older adults receiving care may perceive the presence of a healthcare provider in their home as intrusive or threatening, especially when the care involves medical equipment. Much of the work performed by home care staff involves interactions between the person receiving care (hereafter, also termed service users or users), their relatives, and health and social care workers. The quality of these interactions is an important part of the care of those involved and their general well-being. In some cases, these interactions become complicated and are experienced as difficult by the staff, characterized as frustrating and in some cases leading to feelings of insufficiency. (Larsson Gerdin et al., 2021). This has implications for both the quality of care for service users and the sustainability of the workforce (Brodtkorb et al., 2015; Eilertsen & Kiik, 2016; Falkenstrom, 2017; Larsson Gerdin et al., 2021; Michaelsen, 2021). Although this situation has been studied to some extent, a better understanding of the experience of complicated interaction and how they can be improved in home care services is called for.

### Complicated Interactions

Although complicated interactions have been discussed in nursing for decades, they remain understudied. This may be related to the danger of stigmatizing users and their caregivers. In the 1960s and 1970s, patients or users were commonly considered the root of the problem if interactions became complicated. They were often labeled as “difficult” and became “unpopular” (Michaelsen, 2021; Podrasky & Sexton, 1988; Stockwell, 1972). Over the following decades, such labeling by healthcare workers became unacceptable, and was considered negative and disrespectful. In the 21st century, more emphasis has been placed on exploring the nature of the interactions between all parties involved (Macdonald, 2003; Rozel, 2018; Wolf, 2010).

Experiences of complicated interactions among home care workers have been related to several issues and conditions apart from the care receivers. Pressure to implement cost-containment and efficiency strategies have led to emotional dissonance for staff (Debesay et al., 2014; Graff & Vabø, 2025; Karimi et al., 2017) and has transformed care work in home care from holistic care to task focused care (Ring et al., 2024). Furthermore, time constraints can impact staff communication with users, especially when they face language barriers or come from different cultural traditions (Debesay et al., 2014; Eilertsen & Kiik, 2016).

 Studies have also shown that staff may experience rejection when patients do not accept the care they are offered, or when they do not follow advice (Dobrina et al., 2020). This may occur when staff feel that patients neglect themselves and do not follow expert recommendations for taking care of themselves (Dobrina et al., 2020). In some situations, staff find themselves in between users and their relatives, for instance where relatives call for more service while users want less (Brodtkorb et al., 2015). In such situations the staff experience frustration between wanting to respect the user’s wishes and seeking to understand the relatives’ perspective.

 Interactions may be experienced as difficult in situations that provoke complex emotions among the staff. Such emotions may arise in relation to pain among users that is difficult to control or a life-threatening disease. Staff may feel uncomfortable and helpless when they reflect on their own vulnerability (Dobrina et al., 2020). Research has also shown that in some cases staff feel provoked by users whom they consider demanding, controlling, critical or time consuming, and may acknowledge that not all encounters with users go well (Eilertsen & Kiik, 2016; Falkenstrom, 2017; Koekkoek et al., 2011). Staff may also regard users’ illnesses as self-inflicted, especially if their health has deteriorated due to alcoholism (Karlsson & Gunnarsson, 2018). Researchers have found that staff may resort to, sometimes unhelpful, coping strategies, such as avoiding caring for the user by asking a co-worker to do it, by shutting off, or by emotionally distancing themselves from the situation (Graff & Vabø, 2025; Leppänen, 2008; Michaelsen, 2021).

### Theoretical Background

As the above review shows, experiences of complicated interactions between staff and home care users may influence staff negatively and lead to difficult emotions. The term emotional labor was defined by Arlie R. Hochschild in 1984, later updated 2012 and is commonly used in studies of difficult work situations. Emotional labor captures the effort involved in managing feelings that arise at work. Hochschild points out that the strain involved in emotionally demanding situations may become overwhelming and in worst cases it may affect staff’s health and well-being and hence their work ability (Hochschild, 2012).

Gray (2012) noted that; “emotions are pivotal to understanding, both about ourselves and the caring relationships made in social worlds with others” (p. 1). Effective emotional management is therefore essential to avoid feelings such as hostility or mistrust toward patients. Emotion work involves the conscious regulation of emotions to accomplish tasks, maintain professional boundaries, and influence interactions with others (Graff & Vabø, 2025). It also includes suppressing internal responses such as frustration and anger when they arise. Building on this understanding of emotion work, our research focuses on what Theodosius (2008) describes as “therapeutic emotional labor,” which encompasses the interactions and interpersonal relationships between staff, service users, and their families.

In addition to the theory of emotional labor we drew on authors who have theorized health care as a care practices approach making the social and material interactions central to understanding how care is accomplished (Mol et al., 2010). By exploring how care is enacted in relations (Mol, 2008) these authors are primarily interested in what happens in everyday care situations, drawing our attention to the affect as an effect produced through care practices. In this understanding care practice is understood as engagement among different participants in trying to find out what might be helpful in situations of caring (Bjornsdottir, 2018). Attending to the emotional impact of these situations is of central importance.

In this article, we present findings from the first phase of a research study designed to deepen understanding of why interactions between staff, users, and relatives in home care services can become complicated, how staff experience these interactions, and how practice might be improved to address these challenges. A subsequent article will report the perspectives of users and relatives. The present study was guided by the following research questions: (a) How do home care staff members describe complicated interactions? and (b) How do they experience such interactions?

## Methods

### Design

This was a prospective ethnographic study where events were followed as they unfold, thereby generating insights that may inform and shape future developments. Ethnography focuses on the meanings and consequences of human actions and institutional practices (Hammersley & Atkinson, 2019) and allows for an exploration of practice in context. Using an ethnographic approach allowed us to observe everyday practices in home care as understood by different players. We used both observational and narrative approaches to capture each participant’s story and understanding of the situation (De Fina & Georgakopoulou, 2019; Mishler, 1995). Studying situations characterized by complicated interactions over time allowed us to observe the different ways in which participants responded to these situations, and thus, compare ways that were helpful with those that seemed less helpful (Pols, 2015). Critical incidents, moments of intense emotional experience that usually reflect a breakdown of what is considered normal were particularly important (Gherardi & Rodeschini, 2016).

### Setting, Sample, and Recruitment

The study was conducted in Reykjavik, the capital of Iceland where comprehensive welfare services apply to all citizens like in the other Nordic countries, while maintaining cost containment strategies. At the time of the study, the population of Iceland was approaching 400,000, with approximately 70% of the population living in Reykjavik and neighboring municipalities. Home care services in Reykjavik are fully integrated, meaning that social services and home care nursing work closely together and are situated in the same locale. These services are free of charge, organized in teams located in designated neighborhoods (Gudnadottir et al., 2017). Home care in the Nordic countries offers services to all citizens in need (Turjamaa et al., 2014). At the time of the study there were 6,388 service users receiving home care services in Reykjavik (Velferðarsvið Reykjavíkurborgar, 2021).

Participants were recruited from home care services in Reykjavík. These services are divided into three geographically distinct areas; each with four to five home care nursing and social services teams that work closely together. Nursing teams comprise a registered nurse (RN) who is the team leader, often one or two RNs, and three to five licensed practical nurses (LPNs). Social service teams comprise a team leader, social services’ assistants, administrative workers, and unlicensed care workers. The service provided by the nursing team included treatments for various health conditions, management of assistance with activities of daily living such as showering, management of medications, health education and guidance directed both to users and their caregivers as well as emotional support. The Social care is integrated with nursing and is mainly responsible for issues related to activities of daily living, such as assistance with dressing and showering, giving pre-dosed medications and preparing meals that meet dietary needs. They accompany users to shop for groceries every 1 to 2 weeks and for walks to keep them company. Social care workers also help with household tasks such as cleaning and laundry. Service recipients primarily receive care for physical health needs and, in some cases, have long-established relationships with care staff. They include both older adults requiring support and treatment and younger individuals living with disabilities or disabling conditions. All participants live independently in their own homes within the community.

A purposive sampling method was used to select participants; homecare users, family members and staff from the three neighborhoods. This was done by approaching managers by phone, followed by email, asking them to identify situations where difficult interaction took place. They contacted nursing team leaders and asked them to identify situations where difficulties had emerged, either through complaints from service users or relatives, or by a staff member. Team leaders who were willing to participate either called their clients and relatives or asked them during visits if they were willing to participate in the study. Other staff members who assisted those clients and were willing to participate were recruited. All participants received an introductory letter identifying the aim of the study, their right to withdraw at any point without explanation, and the organization of the data collection. Six users, five family members, and 24 staff members agreed to participate in the study.

### Data Collection

Data were collected from January 2021 to February 2022 and involved observations of interactions between staff and service users as well as semi-structured interviews with the participating users, relatives and staff. Informed consent was obtained from all participants. Initially, data collection was organized around families identified as cases, which involved observations in the home and interviews with users, relatives, and various staff members. As part of this process, one of the researchers (VE) accompanied care workers during visits, shadowed them, and observed their interactions, body language, and conversations (Gherardi & Rodeschini, 2016). Short notes were made during the observations and later expanded into detailed observational and reflective field notes. These observations had been chosen by the research team based on the literature (see Table 1). In this article we focus on the data related to the staff from home nursing and home care social services.

**Table 1.** Observational Guide.

The researcher joins a staff member on the way to the user’s home where all interactions are observed. On the way, the staff member is asked open questions about how they usually behave on such visits, and what is working well and what is not.
At the user’s home, the researcher monitors the progress of the visit. He participates in informal conversations with all involved and writes down reminders to be developed into field notes.
The researcher observes all interactions, both verbal and non-verbal, between the staff member, the user, and their relatives if they are at their home.
How is the staff member received by the user and their relatives at first?
How is the home? Is it clean and tidy? Look for the small things.
Observe what is not said or done and how the staff member and user are cooperating.
How are they expressing themselves?
How is the staff member behaving and working?
How is the distribution of power in the interaction between the user and staff member?
After the observation, the staff member is asked questions about what they were trying to achieve in the visit and what they think was most important.

Time of observations ranged from 40 to 75 min, with one episode at each home. The average length of interviews was 59 min. The staff were interviewed in a meeting room at their workplace. The interviews were semi-structured and guided by an interview guide developed by the researchers based on previous literature (see Table 2 for examples of the key interview questions). All interviews were conducted after observations. A professional typist transcribed the recorded interviews verbatim.

**Table 2.** Key Interview Questions.

For the staff
What is your job title and your main work?
How long have you worked in home nursing / home care?
How would you express the communication between the staff and users in general?
How would you express your communication with the users and their relatives in general?
- Do you have an opinion on whether complicated interactions can be caused by certain aspects of the situation, such as - the organization of the work? - having support from your co-workers? - prevailing attitudes in the workplace?
Can you tell me about your experience of complicated interaction with a user and/or a relative?
Can you describe how complicated interaction with a user/relative has affected you?
How do you see the communication being improved?
Are there some protocols you can follow during complicated interactions at your workplace?

### Data Analysis

In ethnographic research, data analysis is conceptualized as an ongoing process beginning with the designing of the study and lasting until the results are presented (Hammersley & Atkinson, 2019). As the data collection was completed, all the transcribed data (field notes, diary entries and interviews) were analyzed using systematic text condensation (Malterud, 2012), which involved a four-step thematic analysis of qualitative data across cases: (a) all materials were read to gain an overview and preliminary themes were identified; (b) meaning units, which were text fragments containing information related to the research aim and reflecting participants’ experiences and observed actions of complicated interactions as well as identified values, were sorted, and coded; (c) further condensation of code groups and subgroups was conducted with a focus on participants’ own words/concepts; and (d) the condensate was revised to develop descriptions and concepts concerning complicated interactions and values in home care. An example of the data analysis is provided in Table 3.

**Table 3.** Examples of Data Analysis-Systematic Text Condensation.

Preliminary Themes	Identifying and sorting meaningful units	Condensation	Synthesizing	Final themes
Emotions	“I was beginning to experience disappointment with the user and realized I had no right to feel that.” (Nurse 1)	Feeling negative emotions toward users can make staff feel guilty.	When doing care work, you can feel a range of different emotions that can be difficult to experience.	Frictions and emotional difficulties
The home	“He shouted at me that he would not have a hospital bed in his home.” (Nurse Team Leader 2)	The worker was frustrated because the user did not agree to have a hospital bed in his home.	Complications for staff when a user doesn’t want health equipment in their home.	Home and workplace
Alcohol and drug misuse	“Sometimes, users get very drunk, day after day, and they and their homes are in a terrible state.” (LPN 5)	It can be hard to take care of users who are misusing alcohol or drugs.	Complexity in caring for the user who misuses alcohol and drugs	Problems associated with substance use

Data analysis was led by (VE) and carried out in collaboration with co-authors as an ongoing process throughout data collection, continuing thereafter to support validation of the findings. The sample size and data quality were continuously evaluated throughout the research process. New participants were included in the study until the data were considered rich and provided satisfactory information considering the study aim, sample specificity and theoretical background. The researchers moved between the literature, formulation and modification of the interview and observation guides, recruitment, data collection and analysis.

### Ethical Considerations

This study was approved by the Ethics Review Board of the University of Iceland. Ethical considerations followed the basic principles for research outlined in the Declaration of Helsinki (World Medical Association, 2025). The participants received both verbal and written information about the purpose of the study and were informed that they could withdraw from the study at any time. Each participant provided signed informed consent. Participant details were anonymized. Because of the sensitive nature of this study, all participants had the opportunity to talk to a psychiatric nurse, but none expressed the need to do so. All data were treated confidentially, with only the research team having access to them.

### Rigor and Reflexivity

To enhance the study’s methodological rigor, we followed Lincoln and Guba’s (1985) suggestions for enhancing trustworthiness. This involved continuously evaluating the findings’ credibility and transferability. The analysis was conducted through constant dialog between the authors influenced by findings and their dependability. In addition, by discussing possible biases, motivations and interests, the authors sought to ensure the confirmability of the findings. Data collection over a prolonged period resulted in rich narratives and observations.

The first author (VE) has a MSc degree in nursing as well as a diploma in psychiatric nursing. She has 18 years of nursing experience in home care and was familiar with the setting and context. This was necessary to build trust and collect rich data. The co-authors have extensive experience conducting ethnographic research (JP, KB) and were trained in anthropology (JP, AS), with a focus on values, work ethics, and workplace culture. In addition, one co-author (KB) has substantial experience studying home care nursing practice. The different disciplinary backgrounds in the team have enriched the analysis by focusing on complicated interactions in home care, bringing forth experience, emotional aspects, along with values and work-ethics and culture. Finally, Malterud’s (2012) systematic text condensation helped emphasize the importance of producing substantive and rich data.

## Findings

The staff talked about working in homecare as complex relational work in which one had to adapt to adversity. In their account most interactions go smoothly, although they also did describe situations that were emotionally and ethically challenging. We have identified three themes in the data that reflect complicated interactions from the participants perspective; (a) frictions and emotional difficulties, (b) home and workplace, and (c problems associated with substance use.

### Frictions and Emotional Difficulties

The staff described several instances in which they experienced emotional difficulties that made interactions complicated. Expression of frustration, dissatisfaction or anger from users or relatives, was commonly mentioned as disturbing and these interactions were observed during the field-work. Staff members reacted to that kind of communication in different ways. Sometimes they would be calm and stoic, but in other situations they might become irritated which, as they explained made them feel guilty or shameful. In worst cases they experienced a threat and became scared. During one observation the following was described in the field notes:

The wife of a client expressed frustration to a nurse who was attending to the wounds of the client, saying that she felt they didn’t take good care of the wound-dressing storage, and sometimes didn’t have the right dressings to use. I noticed the nurse getting red in her face and seemed to be irritated. She answered that she would let the team leader know. (Field Note 1)

Instances in which users declined treatment recommendations—such as refusing hospital admission despite staff deeming it necessary—elicited feelings of frustration and guilt among staff, who were concerned that they were failing to deliver adequate care or to sufficiently safeguard the client’s health. A nursing team leader said:

Sometimes users are so sick, for example in the final stages of heart failure, and although we put in more supervision and frequent communication with them, we feel that they could get more help in the hospital, but they refuse to go, and it makes me worried for them. (Nursing Team Leader 2)

The staff felt ambivalent in such situations, considering overruling the client’s autonomy but fearing that it would undermine the client’s trust, especially if they had an ongoing relationship with the person.

Another situation that was also challenging for staff was when users pressed for more service or were constantly phoning even though staff had repeatedly accommodated them concerning service. Such situations had prompted staff to introduce boundaries, for example restricting phone calls to twice a day. One team leader in nursing said: “When users constantly demand more service or are phoning a lot, then it is necessary to introduce boundaries, otherwise things get out of control” (Nursing Team Leader 1). According to staff those instances where they had to introduce boundaries occurred rarely.

A common situation was when an older user refused all help and did not allow staff to enter the home and required a change in visit tactics by the staff. A nursing team leader explained on our way to meet a user that, in such situations, they start the service from a distance by phoning the user and proceed with visits very slowly until trust has developed. As she explained she had started visiting the user we were heading to once a week to do wound care. After several weeks she could extend the visits to three times a week. In her opinion being positive with the service users, rather than meeting them with negativity, is of central importance. This strategy, to gradually engage with users, did not always work with families though.

At times, relatives expect services to be provided as quickly as possible, and in some situations, they give staff a key to allow entry into the home. This can help prevent tension or misalignment in the relationship with the family. As a team leader in social care emphasized, being responsive and open to relatives is vital because staff rely on them to support the care of users:

When the relatives ask for more service for the user, I take a step back and think about where the relative is coming from; he is thinking about what is best for his family member, and I try to do my best for them. (Social Services Team Leader 5)

As these findings reflect, staff members in home care experience interactions as complicated when expectations regarding services do not match. Users may want more service than the staff feel they can offer, or they may reject services. Similarly, relatives may call for more involvement than the user wants. In addition, provoking and demanding behavior was seen as a source of complications raising complex emotions.

### Home and Workplace

The second set of situations staff described as complicated was connected to the house being both a home and a workplace and thus a semipublic space. Tensions could arise when users sought to preserve their privacy, while staff aimed to maintain suitable working conditions for both family caregivers and professionals. For instance, staff might suggest bringing certain technologies to the home, such as a lift or a hospital bed; but, in some cases, this was received negatively by the user. As one user explained in an interview, having a hospital bed in the home would give it an institutional look. Although the staff was often sympathetic to those views, the situations nevertheless made their work environment very difficult.

In another case, an older woman living in a large house with two floors, was no longer able to use her upstairs bedroom because of her deteriorating health. She had a bad wound on her sacral area, which needed to be changed daily. The woman slept on a couch in her living room and the nurses had to change dressings while she was lying on the couch; to do this they had to bend to perform wound care. This woman was at risk of developing bedsores and needed a special mattress, while the nurses required a more ergonomically safe arrangement for doing the wound care. Yet the woman resisted all suggestions for having new equipment brought in. As she explained, there was beautiful furniture in her home, including a piano that she used to play; and she was not prepared to change her home into a hospital. This situation represented a common dilemma faced by the staff. They would participate in staff meetings where worker health and safety was emphasized, while being confronted with objections to such arrangements in actual care situations in the homes.

Another issue concerning working conditions for the staff, repeatedly brought up in conversations, was that the cleanliness in homes could be in all kinds of states. At times, staff found they were entering situations where it became immediately apparent that the user had been unable to manage fundamental activities of daily life and domestic activities for an extended period, resulting in a hazardous and unhygienic environment. One staff member described an example of such conditions:

One time, I went to a home of a man who was starting with home care, and he had frontal brain injury and couldn’t take care of himself or his home. I couldn’t get around in the flat because of all kinds of junk everywhere, and when I opened the fridge, there were flies there; this was a very bad situation. Before we could start the service, we had a special team come in and clean the home (Social Services Team Leader 1).

Situations like this were common in some homes, where clutter, including bottles and cans on the kitchen floor, made it difficult for staff to move around. The accompanying nurse noted that this was a frequent observation in the home we visited and in others. These situations raised feelings of helplessness and despair among the staff. Some staff took the stance that the person had brought it on themselves, while others wondered how the user could be helped. In those situations, the staff had to make an agreement with the user or a relative concerning cleaning the home and having permission to do that.

### Problems Associated With Substance Use

Excessive alcohol or narcotic use among service users emerged as one of the most complicated situations in home care services. Sometimes, the staff, although accounting for taking good care of the service users, drawing on values of non-judgmental care, felt that they were just checking on them, to see if they were still alive and not susceptible to harm. Over time often these service users’ health deteriorated, and the staff felt that things did not change for the better, and in few instances the service users died from substance related problems. The staff wondered if they were enabling these service users in their substance use by taking care of them and felt disturbed by these thoughts.

In addition, the staff found it difficult to handle situations when service users who were misusing narcotics expressed substantial pain that could not be mitigated by medication due to the interference with the narcotics used. That was confirmed during one observation, where the service user expressed increased pain, and asked for more pain medicine, although he was getting a full dose three times a day. Staff explained that he often appeared overly sedated, which contributed to feelings of uncertainty and inadequacy in their care. Afterward, the nurse talked about a care plan intervention formulated by the team, where there was a clear message that the staff were forbidden to give him an extra dose of this medicine. Staff reported that the healthcare system had limited resources to address these complications, particularly for service users with active addictions, and that the system did not function well in supporting everyday life. A nurse explained:

I, as a nurse need to remind myself not to be too harsh and unfair toward people who are demanding and abuse drugs or alcohol. Like one user, a man that provokes me a lot of the time, he is pushing boundaries, wants us to come when it suits him, and is phoning a lot. It is of course best to have as few preconceived ideas as possible. (Nurse 1)

As one participant, a nurse who was also an alcohol misuse counselor, explained, it is important for staff to realize that often people who were actively consuming excessive amounts of alcohol or drugs, feel ashamed and find it difficult to trust staff. Therefore, it is important to approach them with respect to establish trust, a simple but not an easy task.

This last theme reflects a particular situation that many participants brought up and described as increasingly common and complicated. Such situations called for opportunities for the staff to talk about instances in which they experienced emotional turmoil related to demanding situations such as the ones described here. They felt that the team leaders could release them from the responsibility of judging what was the right thing to do. In their opinion, team leaders should provide staff with guidance on managing complex situations involving substance use and ensuring safety, such as deploying two staff members when conditions in a home are particularly challenging.

## Discussion

This study aimed at increasing understanding of what makes interactions between staff, users, and relatives in home care services complicated, how the staff experience such interactions, and how home care practice has developed to address them. The study’s substantive contribution to the field provides insight into relational complications and their effects on staff. The findings highlighted the emotional effects and moral dilemmas that participants experienced when interactions become complicated, as reflected in the first theme, *“frictions and emotional difficulties.”* These difficulties were related to establishing realistic expectations toward the services and gaining user trust so that services could start. Some staff experienced frustration and guilt when users did not want any service, and in some cases, they resorted to a coping strategy like displaying irritation and adopting a rigid approach toward users such as rules around the number of calls (Graff & Vabø, 2025). Practices for engaging with users who refused services were complicated by competing family demands, which at times led staff to compromise their professional instincts or judgment. In line with that, Brenne et al. (2024) emphasize that flexibility is essential in home care to exercise professional discretion as unforeseen events cannot always be managed within standardized procedures.

However, some of the staff had developed practices where they would try to develop rapport, hoping that trust would develop, leading to an opening so that they could start to attend to the needs of the user. These situations called for negotiations and demanded that the staff tried to understand what motivated the user’s behavior. Similar experiences have been described in other studies on the importance of open and dignified communication with users and their relatives and to give time in the encounter as not to compromise the quality of care (Brodtkorb et al., 2015; Martinsen et al., 2018).

 The second theme, “*home and workplace*” reflects how complicated things can become when users’ home, which is their private space, is also a workplace for the people who are helping them. Poor working conditions can be challenging for staff. Meanwhile, having a hospital bed at home may change the user’s vision of their home. This situation calls for dialogue and collaboration between the user, relatives and staff in exploring ways to move forward. The word tinkering has been coined to reflect such work (Mol et al., 2010), where different methods are tried out in search of a good solution. For example, an approach to safely perform wound care without altering a person’s home may be to use a portable bench that can be folded and stored away in a convenient place. When working with users whose homes lack cleanliness or who exhibit hoarding tendencies, staff must rely on diplomatic skills to negotiate compromises that support both the user and the provision of care. Such interventions require organizational backing, along with adequate support for staff to carry out this care effectively (De Veer et al., 2022).

The third theme, “*problems associated with substance use*,” highlighted that excessive alcohol or substance use was a recurring and particularly demanding challenge for staff. Caring for patients with alcohol use disorder has been described by others as challenging and complex, as staff may experience feelings of despondency and resignation that can impact the quality of care (Bové et al., 2020). Staff perceptions of being insufficiently qualified to provide necessary care, such as preventive interventions for alcohol use, due to competing work priorities and a lack of training and support have also been reported (Bareham et al., 2020). Staff can adopt reflective approaches, considering how to support the user in leading a meaningful life and using their time productively, while evaluating whether their actions are beneficial or potentially enabling harm (Pols, 2023). They may also discuss strategies for balancing support with boundaries to promote the user’s autonomy and well-being.

 The theory of emotional labor (Hochschild, 2012) guided the analysis, illustrating how the emotional demands of complex interactions with users and relatives can generate negative feelings in staff, including frustration, guilt, and anger. It shows the need for staff to be aware of their emotional expressions and manage their emotions to care for themselves and their service users (Delgado et al., 2017; Theodosius, 2008). This may include critical discussions about whether their interventions are contributing positively to the user’s well-being or inadvertently enabling harm. In navigating such complexities, the professional values they uphold—such as the principles of beneficence and non-maleficence—guide their decision-making. For example, building trust over time can serve as a means of enacting these ethical commitments (Hertzberg et al., 2024). Another form of care action is harm reduction interventions where ways to reduce the negative consequences of substance use are discussed with the user. According to Nixon and Burns (2022), these actions can enhance respect and trust between users and staff members.

This study highlights the complex effects of challenging interactions on staff and underscores the need to provide opportunities to process difficult emotions and reflect on appropriate responses. To reduce stress, staff require guidance in recognizing and understanding their emotions (Delgado et al., 2017; Smith, 2012) and in managing them constructively for the benefit of all involved. An awareness of how they feel when caring for users and how they can manage their feelings in a sound way for themselves as caregivers should be the goal. This calls for acknowledgment of the complex and demanding work involved in home care interactions, the potential for this work to become complicated and the need for organizational and educational interventions that can support this particular kind of nursing and social care work.

### Strengths and Limitations

Using an ethnographic approach, this study allowed us to get closer to the participants while studying the nature and impact of complicated interactions. The findings provide in-depth insight into the situations encountered by home care staff and offer important leads regarding how such situations may be addressed. Given the nature of the data collection, the interpretations presented here are not intended to be generalizable or to provide a definitive understanding of the complex interactions in home care. However, the findings will hopefully provide insights into the further development of communication in home care services. Because the interviewer was a home care nurse herself, there was a risk of assumed knowledge, which the interviewer was aware of and endeavored to reduce. These findings underline the need to develop caring practices for integrated home care services that aim to prevent and address complicated interactions.

## Conclusion

Home care practices are largely relational and complicated interactions in home care can substantially affect the quality of care and the health and well-being both of users and staff if they are not dealt with constructively and holistically. The findings from this study show that staff need to be aware of how they communicate with users and their relatives, and that the relational nature of the work requires adequate time allocated. It is also important for staff to be aware of their own emotions to be able to deal with complex interactions at work.
